# Complete chloroplast genome of *Ulva compressa* (Ulvales: Ulvaceae)

**DOI:** 10.1080/23802359.2020.1860696

**Published:** 2021-03-11

**Authors:** Lihua Xia, Yutao Qin, Jinlin Liu, Haofei Zhang, Lingjuan Wu, Song Gao, Minmin Zhuang, Jing Xia, Shuang Zhao, Yang Xu, Meilin Fu, Yuqing Sun, Yichao Tong, Jianheng Zhang, Peimin He

**Affiliations:** a East China Sea Environmental Monitoring Center, State Oceanic Administration, Shanghai, China; b Ministry of Natural Resources, Key Laboratory of Marine Ecological Monitoring and Restoration Technology, Shanghai, China; c College of Marine Ecology and Environment, Shanghai Ocean University, Shanghai, China; d North China Sea Marine Forecasting Center, State Oceanic Administration, Qingdao, China

**Keywords:** Ulva compressa, green tide macroalgae chloroplast genome phylogenetic analysis

## Abstract

*Ulva compressa* is one of the causal green macroalgae in many countries. In this study, complete chloroplast genome sequence of *U. compressa* was reported, and the total length of this species was 94,226 bp (GenBank accession number MT916929). The overall base composition of chloroplast genome was A (37.2%), T (37.0%), C (12.7%) and G (13.1%), and the percentage of A + T (74.2%) was higher than C + G (25.8%). *U. compressa* chloroplast genome encoded 90 genes, including 63 protein-coding genes, 23 transfer RNAs genes, and 4 ribosomal RNAs genes. The maximum likelihood phylogenetic analysis showed that *U. compressa* is the closest sister species of *U. linza*. This study will be helpful to understand the genetic diversity of *Ulva* species.

China has the high-frequency outbreak of the green tides (Smetacek and Zingone 2013; Hu et al. 2014; Zhang et al. 2014; Zhang et al. 2015; Cui et al. 2019; Liu, Zhao et al. 2020; Liu, Zhuang, et al. 2020; Kang et al. 2020; Xiao et al. 2020). The formation of the green tide in the Southern Yellow Sea of China is complicated. Many researches demonstrated that the significant amount of attached *Ulva* species on the *Pyropia* aquaculture rafts were considered as the main source of the blooms in the Yellow Sea (Liu et al. 2009; Hu et al. 2010; Huo et al. 2015; Zhang et al. 2017; Zhang et al. 2019; Shan et al. 2019; Wang et al. 2018; Zhao et al. 2019; Zhao et al. 2020). The attached *Ulva* species consisted of four species which were *Ulva compressa*, *Ulva linza*, *Ulva flexuosa* and *Ulva prolifera*.

Our laboratory had studied the chloroplast genome of *U. flexuosa* (NC035823) (Cai et al. 2017), *U. linza* (KX058323) (Wang et al. 2017) and *U. prolifera* (KX342867) (Jiang et al. 2019) before. In order to study the chloroplast genome of *U. compressa*, we collected *U. compressa* from the estuary of Nantong, China (32°49′42″N, 121°19′05″E). The specimen was stored in the herbarium of Shanghai Ocean University Museum (SHOU2020NT032202). We sent the specimen to Sangon Biotech (Shanghai) Co., Ltd. for high-throughput sequencing.

DNA of the sample was extracted by using the Dzup (Plant) Genomic DNA Isolation Reagent. The genomic shotgun library was prepared by using the TruSeq DNA Sample Prep Kit (Illumina, USA), paired-end sequences were obtained through the Illumina HiSeq 2500 platform later. *U. flexuosa*, *U. linza* and *U. prolifera* were taken as models for sequence splicing about the complete chloroplast genome of *U. compressa*. We used GeSeq software and Plastid Genome Annotator (PGA) software for annotation (Qu et al. 2019), and used homology comparison for correction, the chloroplast genome was perfectly assembled. Complete chloroplast genome of *U. compressa* was 94,226 bp in length and was annotated in GenBank with the accession number MT916929. The percentage of A + T (74.2%) was higher than C + G (25.8%). The overall base composition of chloroplast genome was A (37.2%), T (37.0%), C (12.7%), G (13.1%), similar to other *Ulva* macroalgae in chloroplast genome. The *U. compressa* chloroplast genome encoded 90 genes, including 63 protein-coding genes, 23 transfer RNAs genes and 4 ribosomal RNAs genes.

In addition, we downloaded sequences from the NCBI database: *Ulva mutabilis* (MK069584), *Ulva ohnoi* (AP018696) (Suzuki et al. 2018), *Ulva* sp. (KP720616) (Melton et al. 2015), *Ulva fasciata* (KT882614) (Melton and Lopez-Bautista 2016), *Ulva lactuca* (MH730972) (Hughey et al. 2019) and *Ulva pertusa* (MN853875) (Han et al. 2020). A Maximum-likelihood (ML) phylogenetic tree with 10 complete chloroplast genome of *Ulva* and 1 outgroup called *Pseudendoclonium akinetum* (NC008114) (Pombert et al. 2006) was constructed by using the MEGA 7 software based on Kimura-2-parameter model (K2P) (Figure 1) (Kumar et al. 2016). This phylogenetic analysis used 1,000 bootstrap replicates to verify the support rate of each node in the tree, and the result showed *U. compressa* was closely related to *U. linza*.

**Figure 1.**
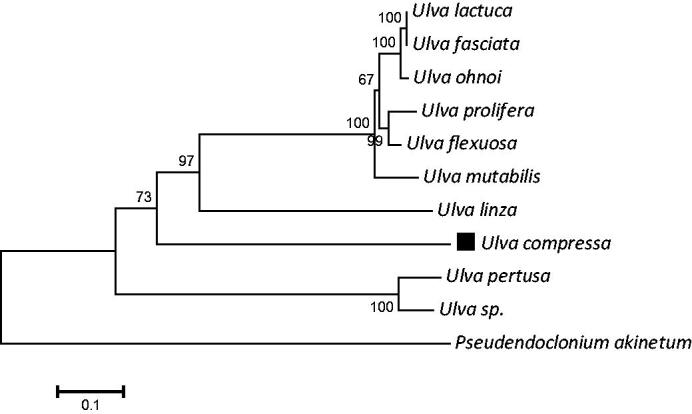
Maximum likelihood phylogenetic tree for *U. compressa* based on the chloroplast genomes. Numbers above each node indicate the bootstrap support value.

In this thesis, we analyzed complete chloroplast genome of *U. compressa* (GenBank accession number MT916929) from the Southern Yellow Sea, which will be useful for studying the genetic diversity of *Ulva* species.

## Data Availability

The data that support the findings of this study are openly available in National Center for Biotechnology Information (GenBank accession number MT916929) at https://www.ncbi.nlm.nih.gov and ResearchGate at https://doi.org/10.13140/RG.2.2.30130.43200.
